# 5:2 intermittent fasting tapers food intake in the refeeding state and ameliorates metabolic disturbances in mice exposed to olanzapine

**DOI:** 10.3389/fpsyt.2022.926251

**Published:** 2022-07-25

**Authors:** Chengfang Zhang, Han Li, Yabin Yan, Xiyan Zhang, Zhilan Tu

**Affiliations:** ^1^Clinical Research Center for Mental Disorders, Shanghai Pudong New Area Mental Health Center, School of Medicine, Tongji University, Shanghai, China; ^2^Clinical Research Center for Mental Disorders, Chinese-German Institute of Mental Health, Shanghai Pudong New Area Mental Health Center, School of Medicine, Tongji University, Shanghai, China; ^3^Shanghai Mental Health Center, Shanghai Key Laboratory of Psychotic Disorders, School of Medicine, Shanghai Jiao Tong University, Shanghai, China; ^4^Department of Pathology, Hospital of Obstetrics and Gynecology, Fudan University, Shanghai, China; ^5^Department of Neurology, Shanghai Pudong Hospital, Fudan University Pudong Medical Center, Shanghai, China

**Keywords:** intermittent fasting, atypical antipsychotics, olanzapine, metabolic disturbances, food intake, satiety hormones

## Abstract

A considerable number of patients suffer from adverse metabolic reactions caused by atypical antipsychotics (AAPs), however, current management strategies are disappointing to clinicians. Preclinical studies have consistently demonstrated that intermittent fasting (IF) has robust disease-modifying efficacy in animal models in a wide range of pathological conditions, especially obesity and diabetes. However, it is unclear what role IF can play in addressing AAPs-induced metabolic disturbances. In our study, we found that a 5:2 IF regimen significantly ameliorated the metabolic disturbances induced by olanzapine (a drug representative of AAPs) in animal models. Meanwhile, our research suggests that IF altering food intake during the refeeding phase may account for the metabolic benefit. This study provides supporting evidence regarding a potentially cost-effective intervention strategy for AAPs-induced metabolic disturbances.

## Introduction

Atypical antipsychotics (AAPs) are first-line treatments for schizophrenia; unfortunately, a considerable number of individuals suffer from the metabolic disturbances it brings, with weight gain as the main clinical manifestation, contributing to significantly higher morbidity from type 2 diabetes and cardiovascular disease in schizophrenic patients (1). Since the underlying mechanism of AAPs-induced metabolic problems is far from elucidated, lifestyle-modification programs are the basis for recent efforts to assist this group in reducing cardiometabolic risks (2). However, lifestyle modifications among schizophrenic individuals are full of challenges, partly due to the poor compliance caused by negative symptoms such as amotivation (3). Another major challenge in the treatment of overweight is the body’s adaptive responses that promote weight regain following energy restriction and weight loss, especially an increased motivation to eat (4). Therefore, although lifestyle interventions aimed at achieving weight loss have been adapted for individuals with severe mental illnesses, analyses show their effectiveness and cost-effectiveness to be disappointing (5). Given the high cardiac risk and premature mortality in this group, strategies for alleviating metabolic disturbances are urgently needed.

Intermittent fasting (IF) is a unique dietary strategy defined as periods of eating alternated with periods of fasting (or severely limited energy intake) that has recently gained much public interest as a lifestyle intervention (6). Studies in animals and humans have demonstrated the benefits of IF in many pathological conditions, especially in metabolic disorders, through mechanisms that extend beyond a reduction in caloric intake (7). Several clinical studies have shown that IF could achieve comparable or even greater improvements in weight and insulin resistance compared to continuous caloric restriction, with superior treatment compliance (6, 8, 9). Moreover, studies using modified fasting paradigms have shown no increase in hunger from baseline during IF interventions (10–12). The advantages of IF appear to fit the needs of those with metabolic disturbances caused by AAPs; however, the effects of IF in AAPs-induced metabolic disorders have not been reported to date.

The three most widely studied IF regimens are alternate-day fasting, the 5:2 diet, and daily time-restricted feeding. In humans, amongst the most favored forms of IF is the 5:2 diet, wherein fasting or severe energy restriction is imposed on 2 days per week with *ad libitum* consumption on the remaining five; it has been shown to be a safe diet pattern in patients with overweight, obesity, and type 2 diabetes (13, 14). Olanzapine is among the most widely prescribed AAPs, but it has been shown to be associated with the greatest risk of metabolic disturbance (1). Therefore, in the current study, we explored the effects of the 5:2 IF regimen on olanzapine-induced metabolic disturbances in a mouse model. The purpose of our research was to investigate whether IF had the potential to be an efficient lifestyle strategy for mitigating AAPs-induced metabolic disturbances.

## Materials and methods

### Animals

**FIGURE 1:**
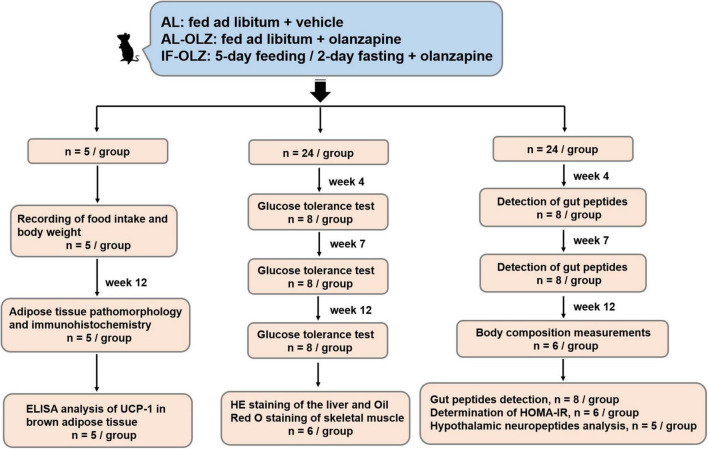
Flow diagram of study design.

The animal experiments were approved by the Ethics Committee for Animal Experiments of Shanghai Pudong New Area Mental Health Center (2018005) and were carried out in accordance with the Guide for the Care and Use of Laboratory Animals of China. Seven-week-old male C57BL/6 mice were purchased from SMOC, Inc. (Shanghai, China). The animals were housed in a specific-pathogen-free (SPF) facility in ventilated cages with controlled environmental settings (23 ± 1°C, 30–60% humidity, 12 h light/dark cycles) and free access to water. All the mice were fed a medium-fat diet (MFD) (metabolizable energy, 17.5 kJ/g), which resembles a typical human diet consisting of 14% protein, 31% lipids, and 54% carbohydrates (15). After a week of habituation, the mice were randomly divided into three groups as follows: AL, AL-OLZ, and IF-OLZ groups. The mice in the AL group were fed *ad libitum* every day and administered 0.9% saline solution as a vehicle by gavage. The mice in the AL-OLZ group were administered 4 mg/kg of olanzapine (Sigma, 132539-06-1, GER) solution by gavage every day with *ad libitum* feeding, as previously described (16). For the IF-OLZ group, olanzapine was administered by the same procedure while subjecting the mice to a 5:2 IF regimen comprising 5-day feeding/2-day fasting periods (Monday and Thursday). During the experiments, body weight and food intake were measured weekly. To evaluate the effect of IF on the refeeding state of mice, tissue and blood samples were collected on the day following non-fasting days. The “resource equation” method is used to calculate the sample size (17). According to this method, the experiment should be of an appropriate size if the degree of freedom of analysis of variance is somewhere between 10 and 20. Therefore, a total of 13–23 animals should be considered as an adequate, and an average of 5–8 animals per group is appropriate. We arranged the experimental procedures based on the consideration of the mutual interference between experiments to reduce the number of animals used, whereby mice (*n* = 53) were divided into three batches. The first batch (*n* = 5) was used to monitor body weight and food intake. At the end of the Week 12 of the experiment, white and brown adipose tissue (BAT) were collected from mice for pathological examination and immunohistochemistry (*n* = 5), and part of the BAT was also used for Enzyme linked immunosorbent assay (ELISA) analysis (*n* = 5). In the second batch (*n* = 24), mice were taken at Weeks 4, 7, and 12 of the experiment for glucose tolerance test (GTT) (*n* = 8). After the end of the GTT operation at Week 12, the liver and skeletal muscle of the mice were collected for pathological examination (*n* = 6). In the third batch (*n* = 24), animals were taken at the Weeks 4 and 7 of the experiment for gut peptides detection (*n* = 8). At the end of Week 12, mice were taken out for non-invasive measurement of body composition (*n* = 6), and then returned to their cages. The blood of the animals was then collected for the detection of gut peptides (*n* = 8), fasting blood glucose and fasting insulin for calculation of the homeostasis model assessment of insulin resistance (HOMA-IR) (*n* = 6), and hypothalamus were subsequently collected for neuropeptides analysis (*n* = 5). The flow diagram of study design is shown in Figure 1.

### Body composition measurements

The total fat and lean mass were measured non-invasively after 12 weeks using a fully automatic Echo-MRI system (Echo Medical Systems, TX, United States) as previously described (18).

### Glucose tolerance test

Mice were fasted for 12 h, and then, their fasting blood glucose was measured using a glucometer (Bayer, Pittsburgh, PA, United States) by tail bleed. Subsequently, mice were administered 2 g of glucose per kilogram of body weight orally, and blood glucose levels were then recorded at 30, 60, 90, and 120 min after glucose administration.

### Enzyme linked immunosorbent assay

Proteins were extracted from interscapular brown adipose tissue (200 mg) by using a homogenizer in radio immunoprecipitation assay (RIPA) buffer (P0013B, Beyotime), supplemented with protease inhibitors (P1005, Beyotime). Then, the soluble fraction was collected by centrifugation at 13000 rpm for 60 min at 4°C to remove cellular impurities and lipids. Protein concentration was determined using the BCA Protein Assay Kit (P0011, Beyotime). In order to compare the UCP-1 content of BAT among the three groups of mice, we quantitatively detected UCP-1 by the ELISA kit (E2067m, EIAab). The analysis was performed following manufacturer’s instructions.

### mRNA expression analysis by qPCR

Mice were intracardially perfused with phosphate-buffered saline; then, the hypothalamus was carefully dissected, and total RNA was isolated using an RNeasy FFPE Kit (Qiagen, Germany). RNA (1 μg) was reverse-transcribed to cDNA using a Reverse Transcription Kit (Invitrogen, United States). Quantitative real-time reverse transcription PCR (qPCR) was performed under standard PCR conditions (50°C for 2 min; 95°C for 10 min; and 95°C for 15 s and 60°C for 1 min for 40 cycles) using the ABI Prism 7900 HT system (Applied Biosystems, United States). Gene expression was analyzed *via* the ΔΔCt method. The primer sequences are shown in Supplementary Table 1.

### Plasma assay

Blood was collected from the iliac vein into an EDTA anticoagulation tube and centrifuged (2500 rpm for 20 min), and the supernatant was collected. Cholecystokinin (CCK), peptide YY (PYY), aglucagon-like peptide 1 (GLP-1), ghrelin (GHR), and leptin (LEP) were detected *via* enzyme-linked immuno-sorbent assays (Cusabio Biotech, CN) according to the manufacturer’s instructions. For the detection of the insulin concentration, mice were fasted for 12 h, and the insulin level was measured *via* a radioimmunoassay. HOMA-IR was calculated as follows: HOMA-IR = fasting insulin (mmol/L) × fasting glucose (mU/L)/22.5.

### H&E, oil red O, and immunohistochemistry staining

All the tissues were fixed in 4% neutral formaldehyde for 24 h at room temperature, followed by dehydration, paraffin embedding, sectioning (4 mm), and staining with hematoxylin and eosin (H&E). To assess the fatty infiltration of skeletal muscle, tissues were snap frozen in liquid-nitrogen-cooled isopentane, sectioned at a thickness of 10 μm with a cryostat, and then stained with Oil Red O as described previously (19).

The procedure for immunohistochemistry was as previously described (20). Briefly, non-specific binding sites were blocked using 1% bovine serum albumin, followed by epitope retrieval using an autoclave (15 min in citrate buffer, pH 6.0). Then, the slides were incubated overnight at 4°C with rabbit polyclonal anti-UCP1 primary antibody (ab234430; Abcam) diluted 1:1000. After the slides were rinsed, they were incubated with HRP-conjugated goat anti-rabbit IgG (HAF008; R&D). Immunovisualization was carried out by the use of diamino-benzidine cytochemistry. The slides were finally counterstained with hematoxylin to display the nuclei. Indirect calorimetry and aerobic testing. Energy expenditure was analyzed using a Comprehensive Lab Animal Monitoring System (Columbus Instruments, United States). Pictures were analyzed using ImageJ (National Institutes of Health, Bethesda, MD, United States). All pictures were assessed by two blinded reviewers.

### Statistical analysis

Statistical analysis was performed using GraphPad Prism5. All the results are expressed as the mean ± standard deviation (SD). The statistical significance of differences among groups was determined by one-way ANOVA plus Tukey’s test. Differences with *P* < 0.05 were considered statistically significant.

## Results

### Metabolic assessments following intermittent fasting

**FIGURE 2:**
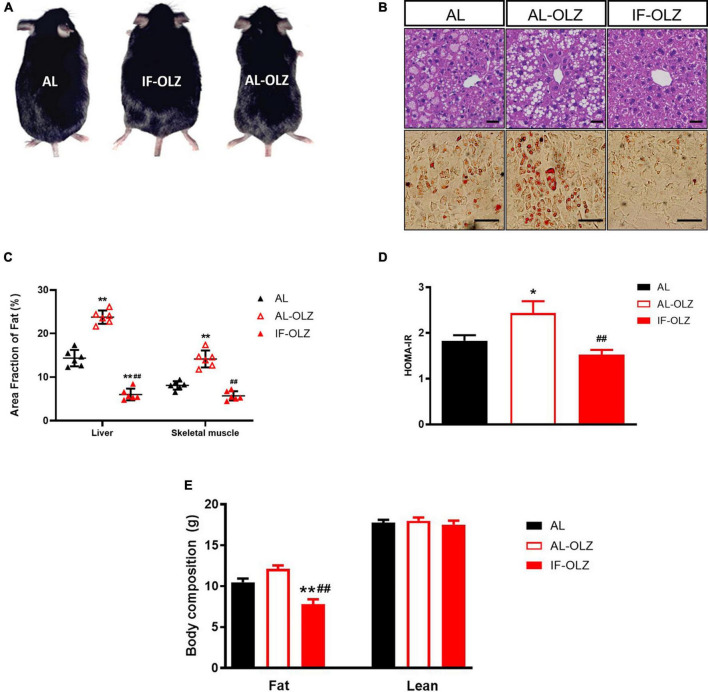
Effect of IF on liver and skeletal muscle steatosis, HOMA-IR and body composition. **(A)** Representative images of AL, AL-OLZ, and IF-OLZ mice. **(B)** Representative H&E-stained sections of liver (upper) and Oil Red O-stained sections of skeletal muscle (lower), scale bars: 50 μm. **(C)** Average area fraction of fat (%) in liver and skeletal muscle were measured using ImageJ software. **(D)** HOMA-IR in AL, AL-OLZ, and IF-OLZ mice. **(E)** Body composition showing fat and lean mass. For each animal group, *n* = 6. One-way ANOVA plus Tukey’s test was performed for the data analysis. Mean ± SD; *P < 0.05 and **P < 0.01 vs. AL; ##*P* < 0.01 vs. AL-OLZ.

Compared to AL mice, the AL-OLZ group exhibited gains in body mass (Figure 2A) and adiposity in peripheral insulin-sensitive organs, manifested as steatosis of the liver and skeletal muscle (Figures 2B,C), and a deterioration in insulin sensitivity as detected by HOMA-IR (Figure 2D). By contrast, the IF-OLZ mice were protected from these effects and even exhibited better metabolic performance than the AL group, suggesting that IF ameliorated not only the metabolic disturbances induced by olanzapine but also the metabolic damage induced by the MFD. Consistently, MRI scans revealed that the AL-OLZ mice had higher whole-body levels of fat accumulation compared to AL mice, while the IF-OLZ group had the least total body fat, and the IF regimen did not result in a reduction in lean body mass (Figure 2E).

### Examination of adipose thermogenesis

**FIGURE 3:**
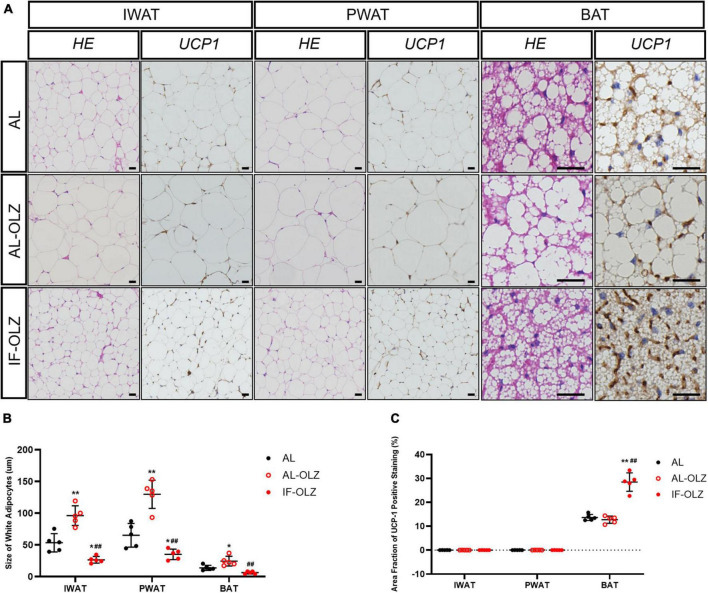
Adipose tissue pathomorphology and immunohistochemistry. **(A)** Representative H&E and UCP1 staining of sections of adipose tissues. IWAT, inguinal WAT (subcutaneous); PWAT, perigonadal WAT (visceral); BAT, brown adipose tissue, scale bars: 50 μm. **(B)** Average adipocyte sizes in mice from H&E images were measured using ImageJ software. **(C)** Average area fraction of UCP-1 positive staining (%) in mice from immunohistochemical images were measured using ImageJ software. For each animal group, *n* = 5. One-way ANOVA plus Tukey’s test was performed for the data analysis. Mean ± SD. *P < 0.05 and **P < 0.01 versus AL; ^##^*P* < 0.01 versus AL-OLZ.

Previous studies have demonstrated that the metabolic benefits brought by IF are primarily attributable to the increased thermogenesis resulting from white adipose (WAT) beiging, especially in subcutaneous WAT (20, 21). We therefore examined the cell morphology and thermogenic function of adipose tissue. We found that olanzapine administration resulted in significantly larger adipocytes compared to AL mice, whereas adipocytes in the IF-treated group were the smallest in size (Figures 3A,B). However, we did not find any beige adipocytes in inguinal WAT (IWAT, subcutaneous) or perigonadal WAT (PWAT, visceral) in IF-OLZ mice, which were characterized by small-volume, multilobular, and dark-colored cell morphology upon HE staining. Non-shivering thermogenesis is mainly mediated by the thermogenic activity of uncoupling protein 1 (UCP1), mainly in brown and beige adipocytes (22). We then detected the expression of UCP1 in both IWAT and PWAT by immunohistochemistry. Consistently, no UCP1-positive adipocytes were observed in the WAT in IF mice (Figures 3A,C). BAT is a major site of adaptive thermogenesis in mice, and BAT activity can significantly contribute to whole-body energy expenditure (23). Although the IF-OLZ group had more area fraction of UCP-1 positive staining compared to the AL or AL-OLZ group (Figure 3C), it should be noted that this may be related to less fatty infiltration in the IF mice (Figure 3B). Therefore, we then extracted total protein and measured the concentration of UCP-1 by ELISA to quantitatively compare the content of UCP-1 among the three groups of mice. However, our data did not show differences between groups (Supplementary Figure 1).

### Food consumption, body weight, and glucose homeostasis

**FIGURE 4:**
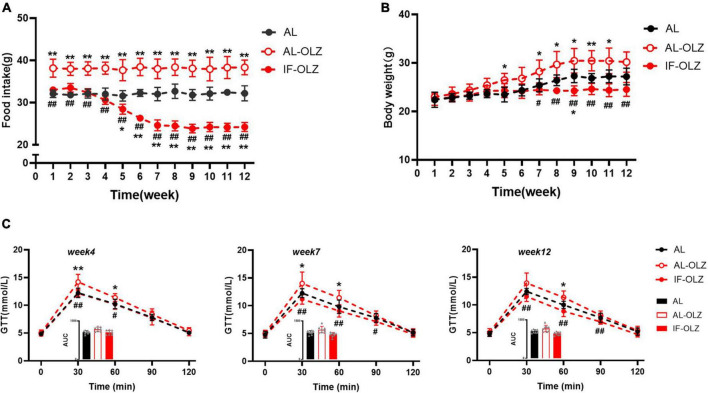
Grouped data showing the relationship of food consumption, body weight, and glucose homeostasis. **(A)** Changes in food intake during 12 weeks of IF cycles. **(B)** Changes in body weight during 12 weeks of IF cycles. **(C)** GTT at Weeks 4, 7, and 12; the inset shows the area under the curve (AUC). *n* = 5 in panels **(A,B)**; *n* = 8 in panel **(C)**. One-way ANOVA followed by Tukey’s test was used for the data analysis. Mean ± SD; *P < 0.05 and **P < 0.01 vs. AL; #*P* < 0.05 and ##*P* < 0.01 vs. AL-OLZ. OLZ, olanzapine.

AL-OLZ mice developed hyperphagia as early as the first week and continued to consume more food than the AL group. However, the intake of the IF-OLZ group showed three stages sequentially with the increase of the IF cycles: comparable to the AL group (1–4 weeks), continued to decline (5–7 weeks), maintained at a level comparable to the AL group (8–12 weeks) (Figure 4A). Considering that the IF-OLZ mice only ingested on 5 days per week, we averaged the weekly food intake of mice to each non-fasting day to observe changes in food intake among the groups (Supplementary Figure 2). We found that the average intake on the non-fasting days in the IF-OLZ mice was significantly higher than that in the AL-OLZ group during the first 4 weeks of the experiment, suggesting that fasting led to compensatory feeding behavior on non-fasting days, which is consistent with the description in the previous research (24). However, food intake in the IF-OLZ group then began to decline, reaching a level comparable to the AL group from Week 7 onward and maintaining that level until the end of the experiment. The above suggests that although IF induces compensatory feeding, it is not sufficient to compensate for the caloric loss caused by fasting. Moreover, as the IF cycle continued to increase, compensatory feeding disappeared and was replaced by a reduction in food intake.

Consistent with changes in food intake, body weight gain was greater in the AL-OLZ group compared to the AL mice. However, the IF-OLZ mice exhibited the flattest weight gain curve, which eventually resulted in a lower body weight than the AL-OLZ group from Week 7 onward (Figure 4B). We further analyzed the effect of IF on the glucose metabolism at Weeks 4, 7, and 12, time points representing three different feeding states. We found that the GTT of AL-OLZ mice was impaired at Week 4, whereas that of IF mice was comparable to that of the AL group, and further improved at Week 7 and Week 12 (Figure 4C). The above data suggest that IF cycles taper the food intakes of mice treated with olanzapine, contributing to metabolic improvements.

### Changes in hypothalamic neuropeptides and circulating gut hormones in the refeeding state

**FIGURE 5:**
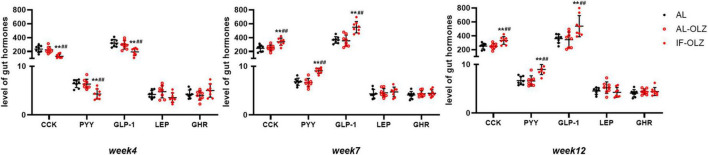
Group data showing the changes in circulating gut hormones. Gut hormones in AL, AL-OLZ, and IF-OLZ mice, *n* = 8. One-way ANOVA plus Tukey’s test was performed for the data analysis. Mean ± SD; **P < 0.01 vs. AL; ##*P* < 0.01 vs. AL-OLZ. CCK, cholecystokinin (pg/ml); PYY, peptide YY (ng/ml), GLP-1, aglucagon-like peptide 1 (pg/ml), LEP, leptin (ng/ml), GHR, ghrelin (ng/ml).

Feeding behavior is directly regulated by central and peripheral appetite signals. Therefore, we detected the mRNA expression of orexigenic and anti-orexigenic neuropeptides from the hypothalamus in the refeeding state. However, hypothalamic neuropeptides, including neuropeptide Y (NPY), agouti-related protein (AgRP), hypocretin (Hcrt), promelanin-concentrating hormone (PMCH), proopiomelanocortin (POMC), and cocaine- and amphetamine-regulated transcript prepropeptide (CARTPT), did not show significant modulation between the groups (Supplementary Figure 3). We further analyzed gut hormones in the plasma in the refeeding state, and we found that IF mice exhibited significantly lower levels of satiety hormones, including CCK, PYY, and GLP-1, at Week 4, whereas their levels were significantly elevated at Weeks 7 and 12. The leptin and ghrelin levels did not differ between the groups (Figure 5).

## Discussion

Previous studies suggested that IF may improve metabolism mainly through thermogenesis caused by WAT beiging, and these studies were performed on high-fat diet (HFD)-induced obesity mouse models with IF regimens of 2:1 (2 days feeding/1 day fasting) or 1:1 (every-other-day fasting), or fasting on 3 non-consecutive days per week (25–27). Interestingly, another study found that the 2:1 IF protocol in the mouse model of leptin deficiency with an ob gene mutation (i.e., ob/ob mice) did not result in detectable WAT beiging, suggesting that the effect of IF is affected by the genetic background and metabolic status (28). In our present work, the phenomenon of WAT beiging was not observed in the context of olanzapine administration with a 5:2 IF regimen. Indeed, IF studies in human clinical settings have reported substantial variability in metabolic benefits, depending on the pathophysiologic conditions of the participants and which IF regimen was employed (7, 29), which suggests that the application of IF needs to be matched to the applicable population.

Several previous animal studies suggested that the cumulative food intake of mice subjected to an IF regimen was comparable to that of the control group due to the compensatory feeding behavior induced by starvation, which became the premise for these studies to speculate that the metabolic benefits of IF are independent of caloric intake (25–27). However, there are also studies showing that IF significantly reduces the total food amount or weekly food intake (30–32). In our present study, the results posit that, although IF-OLZ mice did consume more food on non-fasting days than the AL-OLZ group during the first 4 weeks, this compensatory food intake was insufficient to offset the calorie loss caused by fasting. Unexpectedly, the fasting-induced compensatory feeding in the IF group gradually subsided with increased IF cycles and then continued to decline to levels comparable to AL mice. Under the effect of IF, caloric loss due to fasting and down-regulation of food intake on non-fasting days together resulted in a significant reduction in food consumption in the OLZ group, contributing to the failure to develop a metabolic disorder phenotype. Our findings substantiate the requiring of hyperphagia in olanzapine induced metabolic disturbances (33), and suggest that IF may be an attractive regimen for rectifying AAPs-induced abnormal feeding behavior.

Notably, two studies on the 5:2 IF regimen in obese and overweight populations found that IF resulted in the spontaneous restriction of energy and carbohydrates on non-restricted days (9, 34). Clinical studies have found that alternate-day fasting did not increase hunger as expected, but it increased satiety and decreased food intake on non-fasting days, accompanied by increased circulating PYY (12, 35, 36). A study involving a 2:1 IF regimen in an ob/ob mouse model found that IF induced a significant increase in GLP-1 (37). Our data suggest that the changes in plasma satiety gastrointestinal peptides in the refeeding state are consistent with the metabolic changes with increased IF cycles in mice, suggesting that elevated satiety gastrointestinal peptides may be responsible for IF-induced metabolic improvements. However, the mechanism by which IF induces changes in gastrointestinal peptides under these conditions requires further investigation. The interaction between IF and the gut microbiota has been extensively studied, and the metabolites of the intestinal flora, especially short-chain fatty acids, have effects on the enteroendocrine cells of the gut (38–40), which may be a research entry point worthy of attention. Studies involving 16S rRNA gene sequencing and metabolomic analysis of the gut microbiota, the detection of morphological and functional changes in intestinal endocrine cells, and the validation of the hypothesis by microbiota transplantation may be needed in further research.

Considering that there is currently insufficient evidence to suggest gender differences in the metabolic benefits of IF (41), and nearly all studies have been conducted in male mice in rodent fasting regimens (42), male mice were used in our experiments. However, it is worth noting that the use of only male mice is a limitation of this study, as sex-dependent metabolism difference is commonly observed in both humans and rodent animals (43). On the one hand, sex-dependent effects of olanzapine induced metabolic disorders are seen in some rodent studies. Some studies found that weight gain was only seen in females, while male rodents failed to mimic the clinical situation (44). Although the animal model we used has been proved to be reliable through the detection of various metabolic indicators, however, the use of female mice has the potential to obtain enlarged metabolic effects, and thus making the differences in experimental results even more pronounced. On the other hand, there are major sex differences in insulin sensitivity in intra-abdominal adipose tissue, which is modulated by physiological levels of sex steroids (45), while studies suggest that adipose tissue is also one of the target organs of IF (46). This may imply that the pathophysiological changes produced by IF in olanzapine-treated female mice may differ from those produced in males, that is, the benefits and underlying mechanisms of IF in female animals are both may vary. Therefore, it is necessary to repeat the experiments in female animals to eliminate the bias caused by the sex factor.

In summary, the present work found that the 5:2 IF regimen significantly ameliorated olanzapine-induced metabolic disturbances, probably by the decrease in food intake induced by increased IF cycles. Alterations in the circulating levels of satiety gastrointestinal peptides may underlie the reduced food intake; however, further mechanistic exploration is required. In addition, our study showed that IF-induced compensatory feeding behavior occurred mainly in the first 4 weeks, however, the compliance during this period needs to be verified in the setting of patients taking olanzapine, which will determine whether IF can proceed. This study provides sup-porting evidence regarding a potentially cost-effective treatment for AAPs-induced metabolic disorders.

## Data availability statement

The original contributions presented in the study are included in the article/Supplementary Material, further inquiries can be directed to the corresponding author.

## Ethics statement

The animal study was reviewed and approved by the Ethics Committee for Animal Experiments of Shanghai Pudong New Area Mental Health Center (2018005).

## Author contributions

CZ designed and managed the research. HL and YY conducted animal experiments. XZ and ZT contributed intellectually to data analysis and manuscript editing. All authors have read and agreed to the published version of the manuscript.
