# Supplementary material for: 5:2 intermittent fasting tapers food intake in the refeeding state and ameliorates metabolic disturbances in mice exposed to olanzapine
Source: Front Psychiatry. 2022 Jul 25;13:926251. doi: 10.3389/fpsyt.2022.926251 (PMC9358252; doi:10.3389/fpsyt.2022.926251)
Supplement: Supplementary file 1 [file Table_1.docx]

Supplementary Material

# Supplementary Table

**Supplementary Table 1. Oligonucleotide primers.**

| **Primer Name** | **Sequence (5'–3')** | |
| --- | --- | --- |
|  | **Forward** | **Reverse** |
| Npy | AGAGATCCAGCCCTGAGACA | GATGAGGGTGGAAACTTGGA |
| Agrp | GGCCTCAAGAAGACAACTGC | GACTCGTGCAGCCTTACACA |
| Hcrt | TGGACCACTGCACTGAAGAGA | CAGGGAACCTTTGTAGAAGGAAAG |
| Pmch | AGAGATTTTGACATGCTCAGGTGTA | GGTATCAGACTT GCCAACATGGT |
| Pomc | ACGTGGAAGATGCCGAGATTC | GCACCAGCTCCACACATCTAT |
| Cartpt | TACTGCTACCTTTGCTGGGTG | TTCGATCAGCTCCTTCTCGTG |

Npy: neuropeptide Y; Agrp: agouti-related protein; Hcrt: hypocretin; Pmch: promelanin-concentrating hormone; Pomc: proopiomelanocortin; Cartpt: cocaine- and amphetamine-regulated transcript prepropeptide.

# Supplementary Figures

## Supplementary Figure1


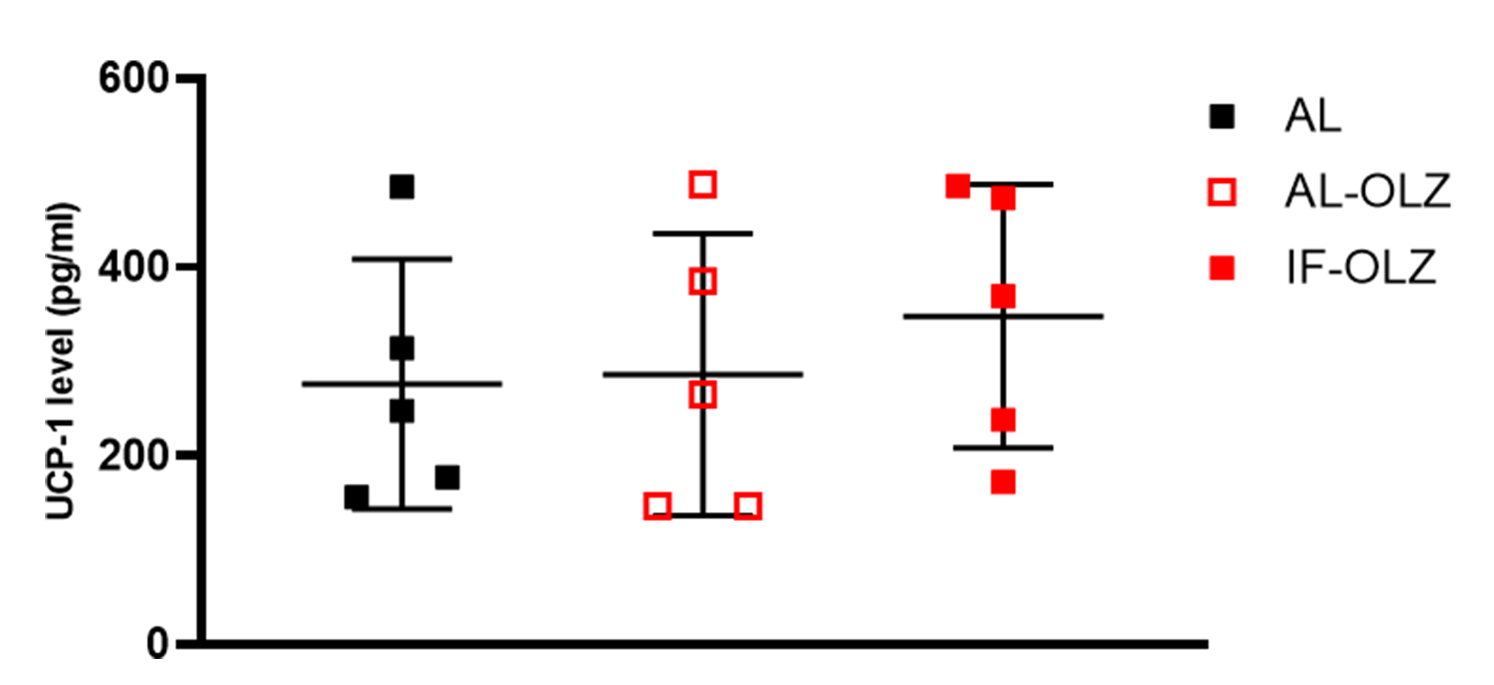


**Supplementary Figure 1.** **Quantitative analysis of UCP-1 in brown adipose tissue by ELISA.** For each animal group, n = 5. One-way ANOVA plus Tukey’s test was performed for the data analysis. Mean ± SD. OLZ, olanzapine.

## Supplementary Figure2
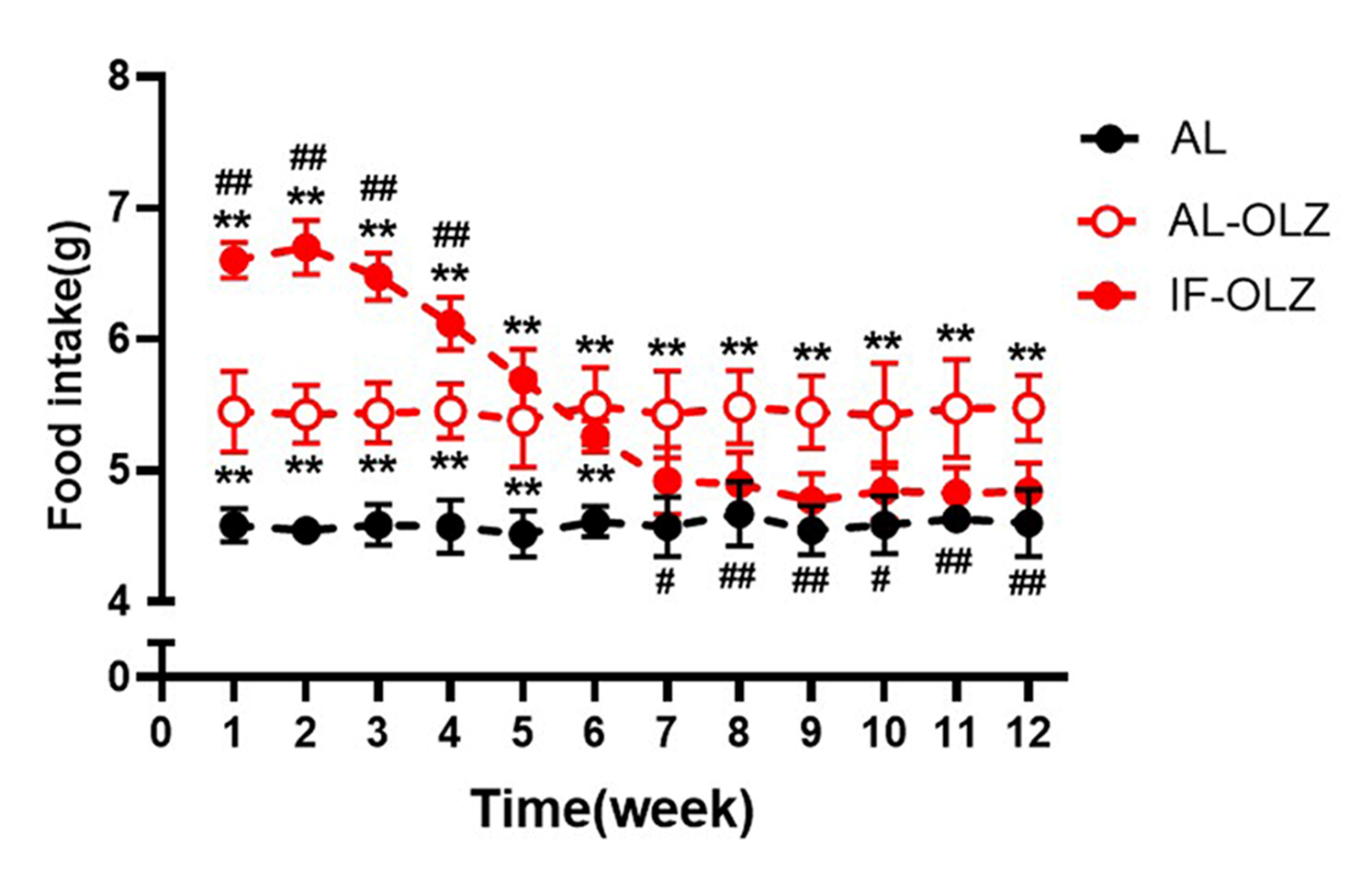


**Supplementary Figure 2.** **Average food intake per non-fasting day.** n=5. One-way ANOVA followed by Tukey’s test was used for the data analysis. Mean ± SD; *P < 0.05 and **P < 0.01 versus AL; #P < 0.05 and ##P < 0.01 versus AL-OLZ. OLZ, olanzapine.

## Supplementary Figure3
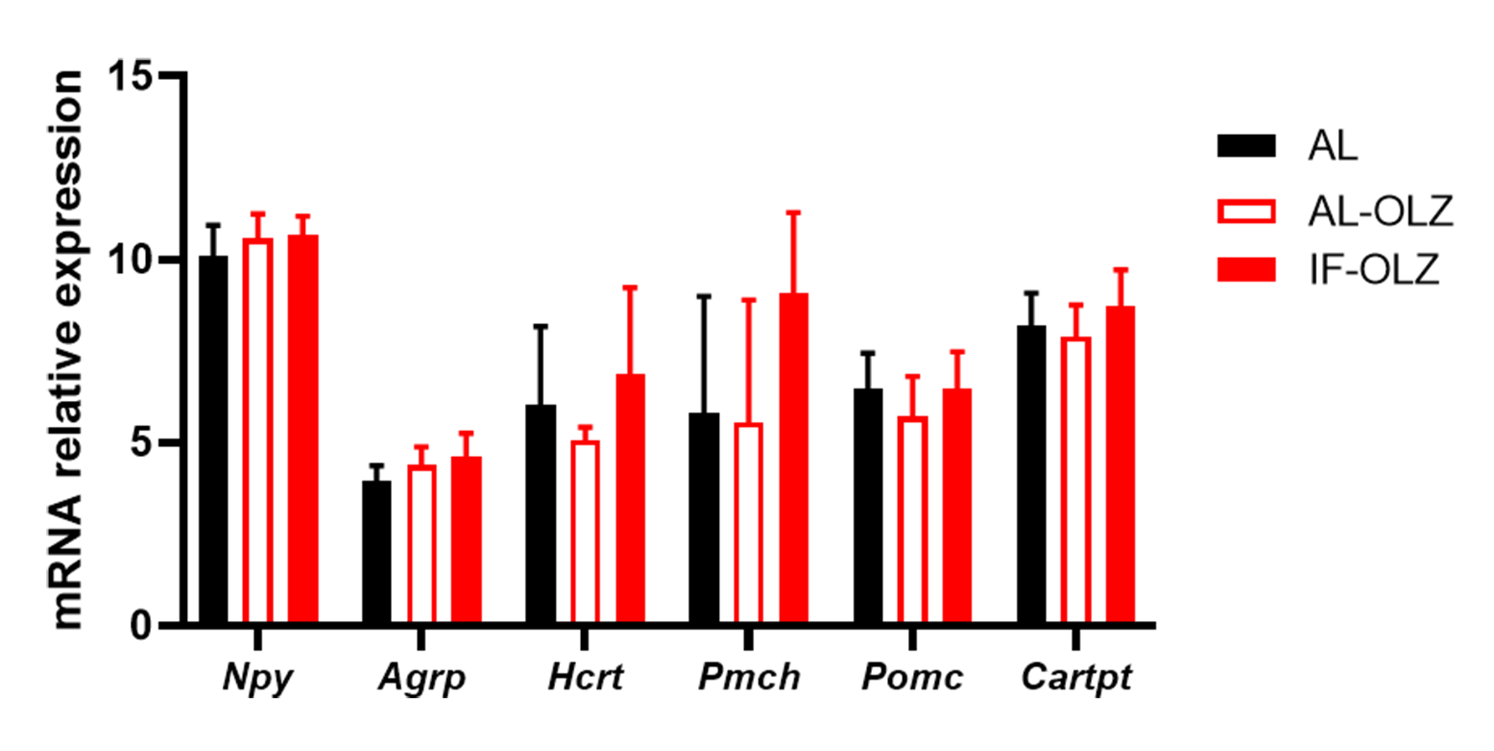


**Supplementary Figure 3.** **The relative mRNA expression levels of orexigenic and anorexigenic neuropeptides of the hypothalamus.** n=5. One-way ANOVA plus Tukey’s test was performed for the data analysis. Mean ± SD; *P < 0.05 and **P < 0.01 versus AL; #P < 0.05 and ##P < 0.01 versus AL-OLZ. OLZ, olanzapine. NPY, neuropeptide; AgRP, agouti-related protein; Hcrt, hypocretin; PMCH, promelanin-concentrating hormone; POMC, proopiomelanocortin; CARTPT, cocaine- and amphetamine-regulated transcript prepropeptide.
